# A high-throughput Sanger strategy for human mitochondrial genome sequencing

**DOI:** 10.1186/1471-2164-14-881

**Published:** 2013-12-16

**Authors:** Elizabeth A Lyons, Melissa K Scheible, Kimberly Sturk-Andreaggi, Jodi A Irwin, Rebecca S Just

**Affiliations:** 1 American Registry of Pathology, 120A Old Camden Rd., Camden DE 19934, USA; 2 Armed Forces DNA Identification Laboratory, 115 Purple Heart Dr., Dover AFB, DE 19902, USA; 3 Present affiliation: Michigan State Police, 7320 N. Canal Rd., Lansing, MI 48913, USA; 4 Present affiliation: Federal Bureau of Investigation, 2501 Investigation Parkway, Quantico, VA 22135, USA; 5 University of Maryland, College Park, 8082 Baltimore Ave., College Park, MD 20740, USA

## Abstract

**Background:**

A population reference database of complete human mitochondrial genome (mtGenome) sequences is needed to enable the use of mitochondrial DNA (mtDNA) coding region data in forensic casework applications. However, the development of entire mtGenome haplotypes to forensic data quality standards is difficult and laborious. A Sanger-based amplification and sequencing strategy that is designed for automated processing, yet routinely produces high quality sequences, is needed to facilitate high-volume production of these mtGenome data sets.

**Results:**

We developed a robust 8-amplicon Sanger sequencing strategy that regularly produces complete, forensic-quality mtGenome haplotypes in the first pass of data generation. The protocol works equally well on samples representing diverse mtDNA haplogroups and DNA input quantities ranging from 50 pg to 1 ng, and can be applied to specimens of varying DNA quality. The complete workflow was specifically designed for implementation on robotic instrumentation, which increases throughput and reduces both the opportunities for error inherent to manual processing and the cost of generating full mtGenome sequences.

**Conclusions:**

The described strategy will assist efforts to generate complete mtGenome haplotypes which meet the highest data quality expectations for forensic genetic and other applications. Additionally, high-quality data produced using this protocol can be used to assess mtDNA data developed using newer technologies and chemistries. Further, the amplification strategy can be used to enrich for mtDNA as a first step in sample preparation for targeted next-generation sequencing.

## Background

Sequencing of human mitochondrial DNA (mtDNA) is performed for a number of purposes in medical, anthropological, population and forensic genetics. In forensics, mtDNA typing is most commonly employed when the nuclear DNA in an evidentiary sample is too limited or too damaged to develop sufficient nuclear data for forensic comparisons. In this application, mtDNA sequencing has historically been limited to the non-coding control region (CR) or portions thereof, where the high concentration of fast-mutating sites presents the greatest opportunity for differentiation of samples representing distinct maternal lineages while minimizing data generation costs and effort. Over the past ten years a number of assays have been developed that interrogate portions of the mtDNA coding region (codR) to resolve maternal lineages which cannot be distinguished by CR typing alone ([1-4], for example), and a very few commercial products are available for the generation of data from the codR. However, the existence of these methods has not yet translated into regular development of mtDNA codR data in most forensic laboratories. The in-house assays developed by various groups are not commercialized, and thus quality control of primers and reagents represents a substantial barrier to implementation; and the commercially-available products are not well-suited for typing the low DNA quantity evidentiary specimens to which forensic mtDNA methods are typically applied [5,6]. Next-generation sequencing technologies may eventually facilitate development of complete mitochondrial genome (mtGenome) data from even very poor quality forensic specimens [7,8]. Yet, before any of these assays and technologies can be routinely applied in forensic casework, complete mtGenome population reference data developed to forensic standards must be on hand to permit generation of the haplotype frequency estimates required for likelihood calculations [5]. At present, no such data is publicly available.

The generation of entire mtGenome haplotypes from even pristine quality and high DNA quantity samples by Sanger sequencing is generally expensive and laborious. A large number of individual sequences are required for sufficient high-resolution coverage across the entire approximately 16.5 kilobase molecule, and past analyses of published mtGenome data sets have identified various errors [9,10]. And while next-generation sequencing technologies are likely to facilitate the development of entire mtGenome data sets, the fact that these methods have not yet been fully vetted and validated for forensic use means that Sanger-based protocols currently remain the only accepted method for the development of complete mtGenome reference data that meet forensic data quality standards [11]. A recently published manual sequencing strategy generates high-quality Sanger sequence data with redundant coverage across the mtDNA codR, and is perfectly suitable for the development of mtGenome reference data when combined with CR sequencing [12]. Yet to ease the way for more rapid, high-volume generation of the complete mtGenome population reference data needed for forensics, accommodate different sample substrates and thus variable DNA quality/quantity, and further decrease the opportunities for human error inherent in manual sample handling, an entire mtGenome sequencing protocol and workflow designed specifically for automated, high-throughput processing is necessary.

To address this need, our aim was to devise a robust amplification and Sanger sequencing strategy that could be used for high-throughput production of complete mtGenome haplotypes which meet the highest data quality expectations while accommodating a wide range of DNA quality and quantity. We report here on the development of an 8-amplicon, 135-sequence mtGenome data generation protocol that was specifically designed to be performed in 96-well format and implemented on robotic liquid handling instruments. The strategy produces redundant sequence coverage across the entire mtGenome in the first pass of automated data generation, and generates high-quality sequences from a range of DNA input quantities and from samples representing diverse mtDNA haplogroups.

## Results

### Assay development

**Figure 1:**
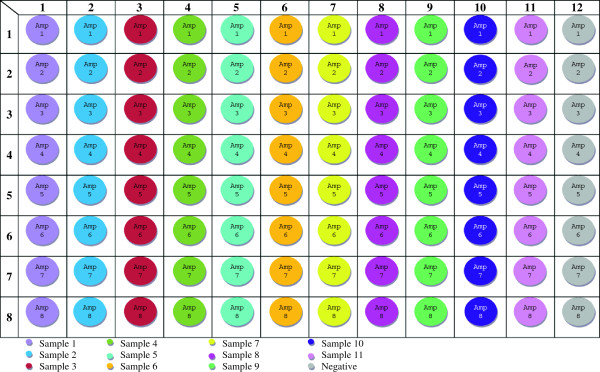
**Amplification plate layout.** Plate map for simultaneous amplification of eleven samples in a 96-well plate. Samples are organized by columns (Sample 1 in column 1, Sample 2 in column 2, etc.), and each row represents one of the eight mtGenome amplicons (Amplicon 1 in row 1, Amplicon 2 in row 2, etc.). Negative controls for each target fragment are amplified in column 12.

Amplification of the full mtGenome in eight fragments was targeted to facilitate sample processing in 96-well plate format, a strategy that permits eleven samples (plus the appropriate negative controls) to be PCR-amplified simultaneously (Figure 1). An established primer set which amplifies the complete CR in an 1198 base pair (bp) fragment [13] was utilized, and the development of seven new overlapping amplicons to span the codR is described below.

Given the need for a robust mtGenome assay that could be applied with equal efficacy across samples representing diverse mtDNA haplogroups, the potential for primer binding site mutations was given careful consideration in the design of codR amplification primers. To this end, a “global alignment” developed from 193 complete or codR only mtDNA sequences was used to assess regions of the mtGenome appropriate for primer placement. The alignment consisted of mtGenome sequences from most major named mtDNA haplogroups (six sequences each from haplogroups A, B, C, D, E, F, G, H, I, J, K, L0, L1, L2, L3, L4, L5, M, N, P, Q, R, R0, S, T, U, V, W, X, Y, and Z; and four and three sequences from haplogroups L6 and O, respectively) sampled at random using GenBank accession numbers available on the PhyloTree mtDNA phylogeny [14]. In addition, published mtGenome substitution rate data drawn from 2196 complete mtGenomes [15] were used to develop a substitution rate histogram by nucleotide position (not shown). In combination, the global alignment and substitution rate graph were used to identify haplogroup-specific mutations and overall highly polymorphic positions and/or regions which could potentially interfere with proper primer annealing during PCR.

Initially, twenty-two codR amplification primers employed for earlier mtGenome sequencing at our laboratory [16-20] were evaluated for use in the new protocol. The global alignment and substitution rate histogram described above were used to assess the potential for primer binding site mutations, and the web-based Primer3 program [21] was used to examine primer characteristics such as melting temperature, GC content, and self-complementarity. Based on the criteria applied all previously used primers were disqualified from further use, most due to potential primer binding site issues. This is not indicative of poor design, but rather reflects the enormous increase in the number of mtGenome sequences available and our general understanding of mtDNA diversity today in comparison to the late 1990s when the prior amplification strategy was initially developed.

Seven new codR amplicons were designed using the global alignment and substitution rate histogram. Within bp ranges deemed acceptable (by virtue of a high degree of sequence conservation, a lack of haplogroup-defining mutations, and sufficient overlap with neighboring amplicons), specific primer sequences were selected using the default settings in Primer3 [21]. Amplification primer sequences are given in Table 1. The average overlap between amplicons is 210 bp, with a minimum overlap of 71 bp (between Amplicons 7 and 8) and a maximum overlap of 338 bp.

**Table 1:** Amplification primers

**Amplicon number**	**Amplicon size**	**Primer name**	**Primer sequence**	**Source**
1	2417	F402	ATCTTTTGGCGGTATGCACTTT	New
		R2818	GCCCCAACCGAAATTTTTAAT	New
2	2381	F2480	AAATCTTACCCCGCCTGTTT	New
		R4860	GAAGAAGCAGGCCGGATGT	New
3	2291	F4609	AAATAAACCCTCGTTCCACAGA	New
		R6899	CATATTGCTTCCGTGGAGTGTG	New
4	2511	F6636	ATTCTTATCCTACCAGGCTTCG	New
		R9146	GCGACAGCGATTTCTAGGATAG	New
5	2489	F8940	CCCCATACTAGTTATTATCGAAACC	New
		R11428	GGCTTCGACATGGGCTTT	New
6	2759	F11319	CAAACTCCTGAGCCAACAACTT	New
		R14077	TTTGGGTTGAGGTGATGATG	New
7	2208	F13835	CAGCCCTAGACCTCAACTACC	New
		R16042	CTGCTTCCCCATGAAAGAAC	[19]
8	1198	F15971	TTAACTCCACCATTAGCACC	[13]
		R599	TTGAGGAGGTAAGCTACATA	[13]

Amplification primer sequences for the eight mtGenome amplicons. The primers for Amplicon 8, which covers the mtDNA CR, were adopted from [13]. The reverse primer for Amplicon 7 (R16042) was previously designed for use as a sequencing primer [19]. All primers except F2480 (Amplicon 2), R4860 (Amplicon 2), R9146 (Amplicon 4) and R14077 (Amplicon 6) are also used for sequencing (see Table 2).

**Table 2:** Sequencing primers

**Amplicon number**	**Primer name**	**Sequence**	**Source**
**1**	F402	ATCTTTTGGCGGTATGCACTTT	New
	F619	TTAGACGGGCTCACATCACC	[19]
	R878	CCAACCCTGGGGTTAGTATAGC	New
	F900	CGGTCACACGATTAACCCAAG	New
	F1135	CCAGAACACTACGAGCCACA	New
	R1136	GGCGAGCAGTTTTGTTGATT	New
	F1320	GACGTTAGGTCAAGGTGTAGCC	New
	R1365	TAGCCCATTTCTTGCCACCT	New
	F1657	CTTGACCGCTCTGAGCTAAAC	[16,17]
	R1769	GCCAGGTTTCAATTTCTATCG	[16,17]
	R1924	AGGTAGCTCGTCTGGTTTCG	New
	F1983	TAGAGGCGACAAACCTACCG	New
	F2105	GAGGAACAGCTCTTTGGACAC	[16,17]
	R2216	TGTTGAGCTTGAACGCTTTCTT	New
	F2333	GCATAAGCCTGCGTCAGAT	New
	R2439	ATGCCTGTGTTGGGTTGAC	New
	F2506	AACATCACCTCTAGCATCACCA	New
	R2818	GCCCCAACCGAAATTTTTAAT	New
**2**	F2625	CTGTATGAATGGCTCCACGAG	New
	F2932	GGGATAACAGCGCAATCCTAT	New
	R3006	ATGTCCTGATCCAACATCGAG	[16,17]
	F3241	AGAGCCCGGTAATCGCATAA	New
	R3417	GGGGCCTTTGCGTAGTTGTA	New
	F3441	ACTACAACCCTTCGCTGACG	[16,17]
	R3632	GAGGTGGCTAGAATAAATAGGAGGC	New
	F3635	GCCTAGCCGTTTACTCAATCC	[16,17]
	R3825	TCAGAGGTGTTCTTGTGTTGTGAT	New
	F3890	GAACCCCCTTCGACCTTG	New
	R4162	TGAGTTGGTCGTAGCGGAATC	[16,17]
	F4142	GATTCCGCTACGACCAACT	New
	F4392	CCCATCCTAAAGTAAGGTCAGC	[16,17]
	R4479	GGGGATTAATTAGTACGGGAAGG	New
	R4676	GATTATGGATGCGGTTGCTT	New
	R4811	TCAGAAGTGAAAGGGGGCTAT	New
**3**	F4609	AAATAAACCCTCGTTCCACAGA	New
	F4925	CCTTCTCCTCACTCTCTCAATC	New
	R5034	ATCCTATGTGGGTAATTGAGGA	New
	F5150	CCTACTACTATCTCGCACCTGAA	New
	R5210	GGTGGATGGAATTAAGGGTGT	New
	F5318	CACCATCACCCTCCTTAACC	[16,17]
	R5325	TGATGGTGGCTATGATGGTG	New
	R5681	GTGGGTTTAAGTCCCATTGGT	New
	F5664	AATGGGACTTAAACCCACAAA	New
	F5858	TTTACAGTCCAATGCTTCACTC	New
	R5799	TGCAAATTCGAAGAAGCAG	New
	R5994	TGCCTAGGACTCCAGCTCAT	[19]
	F6032	GCCAGGCAACCTTCTAGGTA	New
	F6318	CCTGGAGCCTCCGTAGACCT	New
	R6444	TTTGGTATTGGGTTATGGCAG	New
	F6496	CTCTCCCAGTCCTAGCTGCTG	New
	R6899	CATATTGCTTCCGTGGAGTGTG	New
**4**	F6636	ATTCTTATCCTACCAGGCTTCG	New
	F7075	GTATGGGGATAAGGGGTGTA	[16,17]
	R7248	TGGTGTATGCATCGGGGTAGT	New
	F7366	CCTCCATAAACCTGGAGTGA	New
	R7489	TGGCTTGAAACCAGCTTTG	[19]
	F7527	GAAAAACCATTTCATAACTTTGTCA	New
	R7766	TTTCCTGAGCGTCTGAGATGT	New
	F7821	CATCCCTACGCATCCTTTACAT	New
	F8129	ACCACTTTCACCGCTACACG	New
	R8141	CGGTGAAAGTGGTTTGGTTTA	New
	F8355	TTTACAGTGAAATGCCCCAAC	New
	R8378	TTAGTTGGGGCATTTCACTGT	New
	R8640	GATGAGATATTTGGAGGTGGG	New
	F8668	TGACTAATCAAACTAACCTCAAAACA	New
	F8717	AAGGACGAACCTGATCTCTTATACT	New
	R8949	TAGTATGGGGATAAGGGGTGTA	New
	R9031	GGTGGCCTGCAGTAATGTTAG	New
**5**	F8940	CCCCATACTAGTTATTATCGAAACC	New
	F9272	CTCAGCCCTCCTAATGACCTC	New
	R9376	CATTGGTATATGGTTAGTGTGTTGG	New
	F9483	TTCTTCGCAGGATTTTTCTGA	New
	R9611	GGATGTGTTTAGGAGTGGGACT	New
	R9853	GTGAGGAAAGTTGAGCCAATAA	New
	F9832	TTATTGGCTCAACTTTCCTCAC	New
	R10171	TAGAAAAATCCACCCCTTACGA	New
	F10267	CCCTCCTTTTACCCCTACCAT	New
	R10294	AGGGCTCATGGTAGGGGTAA	New
	F10419	AACAAAACGAATGATTTCGACTC	New
	F10689	GGCCTAGCCCTACTAGTCTCAA	New
	R10715	CGTAGTCTAGGCCATATGTGTTG	[19]
	R10942	TAGGGGGTCGGAGGAAAAG	New
	F10950	CCCTCCTAATACTAACTACCTGACTC	New
	R11166	CATCGGGTGATGATAGCCAAG	[16,17]
	R11428	GGCTTCGACATGGGCTTT	New
**6**	F11319	CAAACTCCTGAGCCAACAACTT	New
	F11760	ACGAACGCACTCACAGTCG	[16,17]
	R11768	TGCGTTCGTAGTTTGAGTTTG	New
	R11804	GAAGTCCTTGAGAGAGGATTATGA	New
	F11964	TCACAGCCCTATACTCCCTCT	New
	R12089	TGGGGGATAGGTGTATGAACA	New
	F12194	CCCCTTATTTACCGAGAAAGC	New
	R12302	GCCTAAGACCAATGGATAGCT	New
	F12452	TTGTCGCATCCACCTTTATT	New
	F12741	CAACCTATTCCAACTGTTCATCG	New
	R12766	AGCCGATGAACAGTTGGAATA	New
	R13025	TGGAGACCTAATTGGGCTGA	New
	F13203	AGTCTGCGCCCTTACACAAA	New
	R13390	TGTTAAGGTTGTGGATGATGGA	New
	R13559	GCTCAGGCGTTTGTGTATGAT	New
	F13628	CTAACAGGTCAACCTCGCTTC	New
	R13855	GGTAGTTGAGGTCTAGGGCTGTT	New
	R13924	GGTAGAATCCGAGTATGTTGGAG	New
**7**	F13835	CAGCCCTAGACCTCAACTACC	New
	F14058	CATCATCACCTCAACCCAAA	New
	R14118	TGGGAAGAAGAAAGAGAGGAAG	[16,17]
	F14431	TGCCTCAGGATACTCCTCAAT	New
	R14448	GAGGAGTATCCTGAGGCATGG	New
	F14641	ACCCACACTCAACAGAAACAAA	New
	R14721	CGATGGTTTTTCATATCATTGG	New
	F14881	CACCACAGGACTATTCCTAGCC	New
	R14902	GGCTAGGAATAGTCCTGTGGTG	New
	F15190	CTTACTATCCGCCATCCCATA	New
	R15396	TTATCGGAATGGGAGGTGATTC	[16,17]
	F15500	GACCCAGACAATTATACCCTAGCC	New
	R15585	ATTGTGTAGGCGAATAGGAAATA	New
	F15699	GCCCACTAAGCCAATCACTT	[19]
	R15728	GGAGTCAATAAAGTGATTGGCTTAG	New
	R16042	CTGCTTCCCCATGAAAGAAC	[19]
**8**	F15971	TTAACTCCACCATTAGCACC	[13]
	F16190	CCCCATGCTTACAAGCAAGT	[13]
	F155	TATTTATCGCACCTACGTTC	[13]
	F314	CCGCTTCTGGCCACAGCACT	[13]
	R16410	GAGGATGGTGGTCAAGGGA	[13]
	R285	GTTATGATGTCTGTGTGGAA	[13]
	R484	TGAGATTAGTAGTATGGGAG	[13]
	R599	TTGAGGAGGTAAGCTACATA	[13]

Sequencing primers for the complete mtGenome, with sources (new or previously published). All primers for Amplicon 8, which covers the mtDNA CR, were adopted from [13]; these are used in duplicate to produce a total of sixteen sequences for the CR. Most amplification primers are also utilized as sequencing primers (see Table 1).

Considerations given highest priority in the design of the mtGenome sequencing strategy were 1) the desire to develop high-resolution sequence coverage in both the forward and reverse directions across as much of the molecule as feasible, and 2) a protocol that would be amenable to high-throughput processing on automated liquid-handling instrumentation. For the CR, the sequencing approach described by [13] was adopted. CodR primers previously utilized by our laboratory for mtGenome sequencing [16-20] were evaluated using the global alignment, substitution rate histogram, and Primer3 software [21], as described above. In addition, the typical quality of the sequence data produced by seventy-four of these primers was assessed by inspection of 2237 previously-generated sequence electropherograms, and only primers which routinely produced data with sufficient signal and minimal noise were considered for further use. As a result of these examinations, twenty sequencing primers were maintained for use in the new protocol. Ninety-nine new primers were selected in the same manner as described above for the amplification primers, with old and new codR sequencing primers spaced at intervals designed to produce overlapping, high-resolution forward and reverse sequence coverage across the genome.

**Figure 2:**
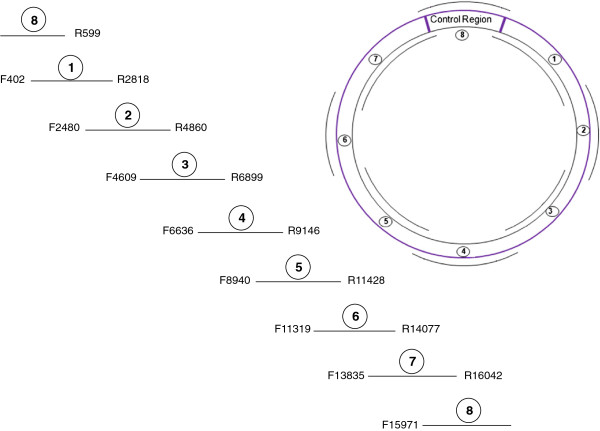
**Amplification of the mtGenome in eight fragments.** Positioning of the eight, overlapping target fragments around the circular mtGenome, along with the primers used to amplify each region, is depicted.

**Figure 3:**
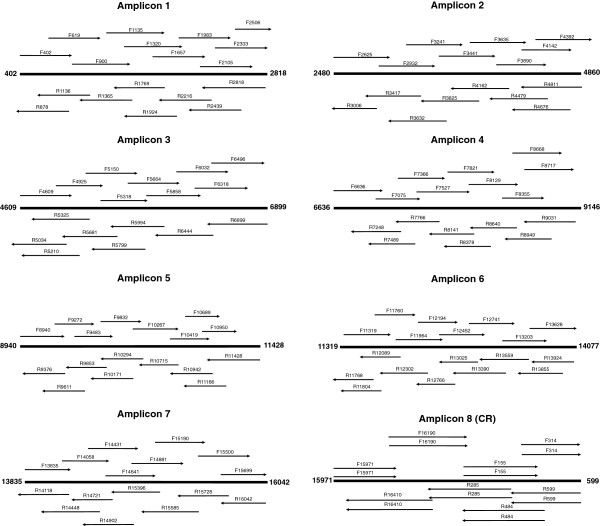
**Organization of sequencing primers by amplicon.** Approximate positioning and coverage of each of the 127 sequencing primers used to generate 135 sequences across the mtGenome. Amplicon start and end points (in terms of nucleotide position) are given, and forward sequences are represented above the template strand while reverse sequences are listed below. The number of sequencing primers per amplicon ranges from sixteen to eighteen. The sequencing strategy used for the CR (Amplicon 8) was adopted wholesale from [13] and uses eight distinct primers to produce sixteen sequences.

**Figure 4:**
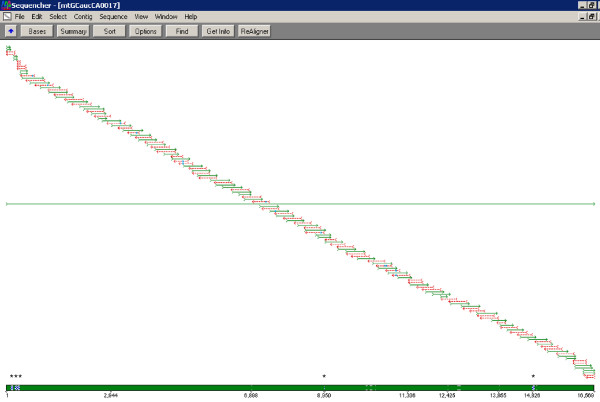
**mtGenome sequence coverage.** This Sequencher (Gene Codes Corporation) screen capture demonstrates the typical mtGenome sequence coverage that results from the 135-sequence strategy. Individual forward sequences are denoted in green, and reverse sequences are represented in red. The data come from a population sample processed for the project described in [26], and no reprocessing was required to achieve complete coverage across the mtGenome. Small regions with replicate but unidirectional coverage (three in the CR due to polycytosine stretches and length heteroplasmy, and two in the codR, totaling 294 bp) are indicated by blue hashing in the coverage bar and asterisks.

The final, 8-amplicon mtGenome strategy is depicted in Figures 2 and 3. The number of sequencing primers per amplicon ranges from sixteen to eighteen, and the strategy produces 135 sequences from 127 unique primers. The resulting redundant sequence coverage across the complete mtGenome is demonstrated in Figure 4. Sequencing primers and their sources (published or new) are listed in Table 3. Thermal cycling conditions implemented for PCR and sequencing are identical to those previously described for complete mtGenome sequencing [16-20], with one exception: as the coding region amplicons in this assay range in size from 2208 to 2759 bp, a 2.5 minute extension time was selected to balance PCR product generation and total thermal cycling time. Thermal cycling details are included in the Methods section.

**Table 3:** Amplicon PrimerBLAST results

**Amplicon number**	**Number of nuclear genome matches**	**Nuclear genome location**	**GenBank accession number**	**Priming region similarity (forward; reverse)**	**Size difference from mtDNA target (in bp)**	**Sequence similarity to rCRS**
1	1	Chr 11 (9510471–9507925)	NT_009237.18	76.2%; 76.2%	170	< 60%
2	1	Chr 6 (53426379–53424135)	NT_025741.15	84.2%; 78.9%	118	< 60%
3	1	Chr 1 (43791–46082)	NT_004350.19	100%; 100%	1	98.47%
4	2	Chr 1 (45818–48327)	NT_004350.19	95.5%; 100%	1	98.57%
		Chr 5 (7704044–7701575)	NT_034772.6	95.5%; 90.9%	1	88.53%
5	2	Chr 7 (6847950–6850445)	NT_033968.6	84.0%; 88.9%	7	75.16%
		Chr 2 (10717704–10720523)	NT_022135.16	80.0%; 88.9%	331	63.40%
6	2	Chr 5 (42577018–42574300)	NT_034772.6	95.5%; 100%	0	94.02%
		Chr 5 (7699358–7696640)	NT_034772.6	90.5%; 90.0%	0	88.97%
7	4	Chr 5 (2218206–2220412)	NT_034772.6	95.2%; 90.0%	1	87.27%
		Chr 5 (2218173–2220412)	NT_034772.6	81.0%; 90.0%	32	87.19%
		Chr 17 (13111672–13109937)	NT_010718.16	80.0%; 85.0%	453	< 60%
		Chr 7 (5831689–5829543)	NT_033968.6	81.0%; 75.0%	41	< 60%

CodR amplification primer pairs were queried against the reference assembly of the complete human genome using PrimerBLAST [24], and results which met specific similarity criteria were noted. For these thirteen regions of the nuclear genome which are potentially amplifiable using the codR PCR primer pairs listed in Table 1, the nuclear genome sequence was aligned to the rCRS [22] to determine a percentage sequence similarity. The two Chromosome 5 matches listed for Amplicon 7 represent slightly different primer binding sites within the same portion of the chromosome.

All steps of the mtGenome protocol described here were designed with high-throughput applications in mind. To this end, plate layouts and programs which permit efficient sample handling and reaction set-up on robotic instrumentation were developed to facilitate highly automated data generation. Details of our high-throughput process, including plate maps and strategies for amplification, sequencing, and purification steps, are covered in the Methods section.

### Sensitivity testing

**Figure 5:**
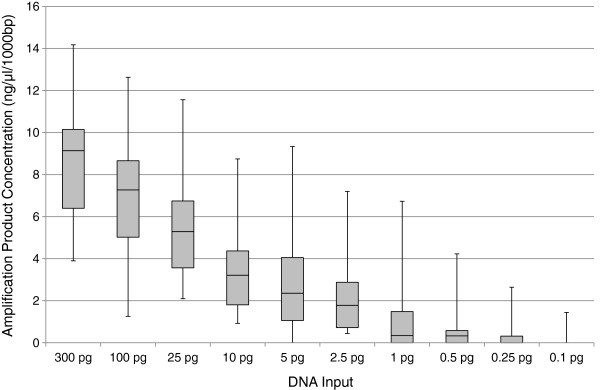
**Amplification sensitivity with positive control DNA.** Results from duplicate amplifications of all eight mtGenome fragments with ten input levels of positive control DNA (9947A). Amplification product concentrations of the target fragments were measured by automated injection on a QIAxcel Advanced capillary electrophoresis instrument (QIAGEN Inc.), and were normalized to ng/μL per 1000 bp (given the variable sizes of the eight target fragments). The wide range of product concentrations represented in the long “whiskers” reflects the differing amplification efficiencies of the eight primer pairs; however, PCR product was produced for all regions down to 10 pg input DNA.

To assess the sensitivity of the amplification protocol, PCR was performed in duplicate for a range of positive control (Human Cell Line DNA 9947A; Life Technologies, Gibco, Carlsbad, CA) DNA input quantities (300 pg, 100 pg, 25 pg, 10 pg, 5 pg, 2.5 pg, 1.0 pg, 0.5 pg 0.25 pg, and 0.1 pg). The PCR products were quantified using the QIAxcel Advanced system (QIAGEN Inc., Valencia, CA) and the resulting values were normalized with respect to amplicon size to enable direct comparison. Figure 5 displays a box and whisker plot of the normalized amplification product concentrations, reported here in ng/μL per 1000 bp, at each DNA input concentration. The long whiskers (highly variable product concentrations) at each DNA input level reflect the range of sensitivities of the eight primer pairs, however all regions were successfully amplified down to 10 pg of input DNA. Beginning at 5 pg input DNA a few amplification failures were observed, and below 1 pg input successful amplification was sporadic and limited to a few high-efficiency primer pairs.

### Developmental validation on population samples

To evaluate the performance of the protocol on a variety of haplotypes, eleven anonymous, high-quality population samples from ten distinct mtDNA haplogroups (A, B, C, D, H, U, K, L1, L2 and L3) were amplified in duplicate and sequenced using the automated, high-throughput process described in the Methods section. The DNA input for PCR varied by sample, and ranged from approximately 0.1-1.5 ng. Trimming and assembly of the raw electropherograms for replicate samples was performed by separate individuals according to laboratory standard guidelines for data quality in terms of background to noise ratio and peak resolution. Sequence coverage across the molecule was assessed in terms of a) redundant and bi-directional coverage, b) the degree of additional manual re-processing that would be required to develop complete replicate coverage, and c) the correlation between sequence coverage and sequence distance from the revised Cambridge Reference Sequence (rCRS) [22]. The final haplotypes for each sample were compared to control data (complete mtGenome profiles previously developed from the same sample extracts [20]).

**Figure 6:**
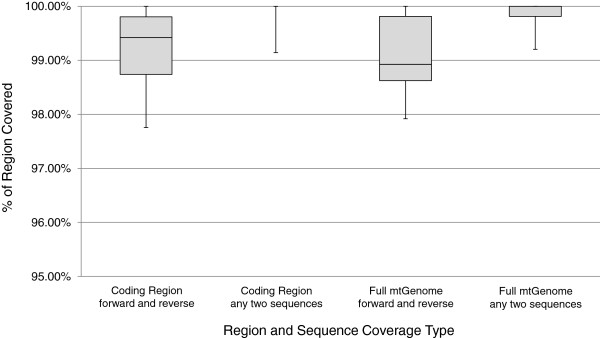
**Sequence coverage.** Percentage of the codR or full mtGenome with redundant sequence coverage following a single pass of automated data generation for eleven population samples, representing a range of mtDNA haplogroups, processed in duplicate. One sample, for which all sequence data in a single direction for a single amplicon was unusable and sourced to instrument failure, was removed from the analysis as an outlier; and small regions of unidirectional sequence coverage due to length heteroplasmy in hypervariable regions one and two in some samples were ignored. On average across the twenty-two samples, high-quality forward and reverse coverage was produced for 99% of the mtGenome.

**Figure 7:**
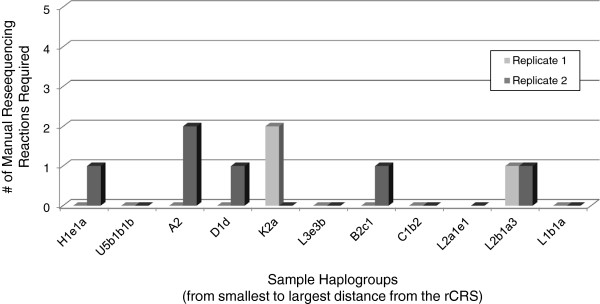
**Reprocessing required.** The number of manual resequencing reactions that would be required to achieve complete double stranded coverage for the twenty-two population samples (duplicate processing of eleven distinct samples) ranged from zero to two. This equates to approximately one resequencing reaction for every two mtGenomes processed. Replicate 1 of the sample representing haplogroup L2a1e1 was not included in the analysis due sequence failures resulting from instrument failure.

High quality sequence data (as defined by signal to noise ratio) was developed from most primers for most samples in a single pass with the automated system. As Figure 6 depicts, on average 99.87% (SD = 0.23%) of the mtGenome was covered by at least 2 sequences, and 99.07% (SD = 0.67%) of the mtGenome had both forward and reverse sequence coverage when small regions with unidirectional coverage due to length heteroplasmy in hypervariable regions 1 and 2 were ignored. The number of manual resequencing reactions that would be required to achieve redundant coverage ranged from zero to two (Figure 7), with approximately one resequencing reaction required for every two complete haplotypes. Considering that 135 sequences were generated for each sample, this equates to a 0.32% resequencing rate. A weak but non-significant correlation was observed between mtGenome coverage and sequence distance from the rCRS, with a mere 1-3% of the variance in mtGenome coverage attributed to sequence distance (data not shown). In all cases the final haplotype matched the haplotype previously developed for each sample.

### Potential for NUMT amplification

Though amplification of nuclear insertions of mitochondrial DNA (NUMTs) is unlikely when sufficient mtDNA is present in a sample [23], the reference assembly of the complete human genome was nonetheless queried using PrimerBLAST [24] for the seven codR amplification primer pairs. Any close sequence matches (defined as 75% or greater overall similarity for both primers, with no more than one mismatch in the 3′ most 5 bp) that could potentially amplify a fragment similar in size to the authentic mitochondrial target (less than 500 bp difference) were further evaluated. For each potentially amplifiable nuclear genome region, the percentage similarity to modern mtDNA was assessed by aligning the NCBI reference sequence to the rCRS in Sequencher version 4.8 (Gene Codes Corporation, Ann Arbor, MI). When the nuclear genome sequence region could not be aligned to the rCRS due to high dissimilarity, the percentage similarity was noted as being less than 60%.

Using the described criteria, thirteen potentially amplifiable regions of the nuclear genome were identified (Table 3). Of these, only three had a sequence similarity to the rCRS greater than 90%. For the two Chromosome 1 regions with greater than 98% sequence similarity to Amplicon 3 (2291 bp) and Amplicon 4 (2511 bp), the Chromosome 1 sequence differed from the rCRS sequence at thirty-five and thirty-six nucleotide positions, respectively. This region in Chromosome 1 corresponds to a described NUMT approximately 5842 bp in length [25]. No NUMT amplification was observed during protocol development or developmental validation.

### Sequencing artifacts

**Figure 8:**
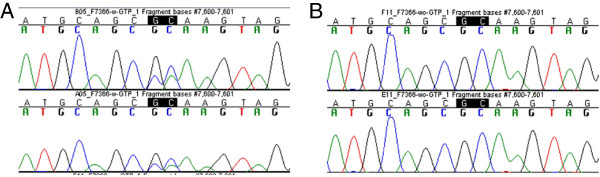
**Sequencing artifacts. A)** Screen captures of forward sequences aligned in Sequencher (Gene Codes Corporation) showing compression and unusual peak morphology around nucleotide position 7600. **B)** When the same primer (F7366) was used without the addition of dGTP BigDye® (Life Technologies, Applied Biosystems), the sequencing artifacts were no longer apparent.

Sequencing artifacts (i.e. small regions of compression and/or unusual peak morphology) due to region-specific sequence motifs were reproducibly observed in both the positive control samples sequenced during protocol development and the developmental validation on population samples. Typically, each artifact was observed in a single sequence direction, and the severity of the artifact varied by primer distance from the artifact. An example of a sequencing artifact is shown in Figure 8.

The standard sequencing protocol used at our laboratory for high-throughput generation of mtDNA population data includes one-quarter the recommended volume of BigDye® Terminator v1.1 Ready Reaction Mix (Life Technologies, Applied Biosystems, Foster City, CA) and replaces 25% of the dITP-containing BigDye® with dGTP BigDye® Terminator v1.1 Ready Reaction Mix ([19]; reaction volumes are specified in the Methods section). The addition of dGTP BigDye® was originally implemented to assist the sequencing of difficult templates, specifically GC-rich regions or polycytosine tracts, in the reverse direction. For this protocol, dGTP BigDye® was eliminated from forward sequencing reactions to reduce the number of artifacts produced in those sequences (Figure 8). All remaining artifacts (nearly all of which occurred in the reverse direction) that were consistent and reproducible across multiple samples, and with replicate sequencing, were cataloged. In practice in our laboratory, this catalog is referenced during assembly and analysis of mtGenome sequences, and known artifacts are annotated in the assembled contig.

It is worth noting here that these types of sequencing artifacts are typically only apparent and recognizable as such because the quality of the sequence data produced is generally pristine. With even a small amount of noise in the sequence data, many of these artifacts would not be evident. In general, the artifacts do not confound data interpretation, as they are typically minor and apparent in only one sequencing direction. Nevertheless, when previously uncataloged artifacts are encountered during data production, our practice is to note the affected bases as ambiguous and resequence the region to confirm that the authentic sequence is represented in the consensus sequence for the region.

## Discussion

Though the mtGenome amplification and sequencing protocol we have developed can be performed manually (with, we must emphasize, abundant attention paid at pipetting steps to prevent sample misplacement), the strategy was specifically designed to be implemented on liquid handling instruments to facilitate high-throughput data generation. In our laboratory, all pre-PCR pipetting steps (including sample placement, extraction and PCR reaction set-up) are performed in 96-well plate format on a benchtop liquid handling robot; amplification product detection is performed directly from the 96-well plate on a capillary electrophoresis instrument; and, with the exception of the addition of enzymes for post-PCR purification (which, due to high viscosity, are pipetted manually into the sample plate to reduce reagent waste and cost), all post-PCR pipetting steps are performed robotically. The particulars of our automated sample processing workflow are detailed in the Methods section.

The high-throughput strategy described here is presently being employed in our laboratory to develop complete mtGenome haplotypes from anonymous blood serum specimens for a National Institute of Justice funded reference population databasing project. Though frequently used for cancer biomarker detection, blood serum is a challenging source for forensic DNA typing as the only DNA present in these samples is residual [26]. Using a silica column based extraction protocol, DNA concentrations (measured using an mtDNA quantitative PCR assay) for a set of 242 blood serum extracts averaged just 15 pg/μL. When those extracts were amplified for the mtGenome, PCR success was strongly dependent on input DNA quantity. Overall, however, the amplification results were consistent with those obtained during sensitivity testing of this protocol, where amplification failures were observed at DNA inputs below 10 pg (see Figure 5). With the blood serum specimens typed using this protocol for the databasing effort, 86.6% of all amplification failures occurred when PCR inputs were less than 10 pg; and at DNA input quantities equal to or greater than 10 pg, 99.4% of amplifications were successful (data not shown).

Based on the observation of some PCR failures with positive control DNA (this paper) and blood serum extracts when DNA concentrations were low, and given the extent of sample reprocessing necessary at various PCR input DNA quantities with the blood serum specimens, we suggest an input DNA concentration for PCR of 50 pg or greater when possible. Further, due to the increased noise (a result of excessive electrophoretic signal) observed in some sequences during the development of this protocol when DNA inputs for PCR were high (data not shown), we recommend that highly concentrated sample extracts be diluted so that PCR input does not exceed 1 ng. Though high quality data has been developed from higher and substantially lower DNA inputs using this protocol, inputs between 50 pg and 1 ng should ensure consistent amplification success and the production of high-quality sequence data across all amplicons in the first pass of sample processing. Following these DNA input guidelines will accordingly reduce the opportunities for human error inherent in manual sample reprocessing and minimize the cost to generate each mtGenome haplotype.

Regarding the potential for amplification of portions of the human nuclear genome (covered in the Results section and summarized in Table 3), it seems highly improbable that a NUMT sequence would be represented in a completed mtGenome haplotype developed using this protocol. Amplification of a nuclear genome sequence alone (in place of the target mtDNA) is extremely unlikely given the abundance of mtDNA relative to nuclear DNA in human cells, and could reasonably only be expected to occur if mtDNA were nearly or completely absent in a DNA extract [23]. In the unlikely case that a NUMT were amplified in place of the mtDNA target, any close inspection of the data (which would reveal an excess number of differences from the rCRS; unusual insertions, deletions, and/or transversions; etc.) or attempt to assign a haplogroup to the mtGenome profile would readily indicate a problem. A more likely scenario with an overall low DNA quantity sample is co-amplification of a NUMT with the authentic mtDNA, which could occur when by chance the mtDNA primers encounter a close-match nuclear DNA target during the early cycles of PCR. While we did not encounter this during protocol development, it is possible that it may be observed as more samples – and particularly those with extremely low DNA template quantities – are processed with this assay. However, if NUMT co-amplification were to happen, it would a) likely occur with only one of the eight mtGenome amplicons at a time, and b) present as a clear mixture in the sequence data for that amplicon, as the high number of positions at which two bases would be observed could not reasonably be explained by mtDNA heteroplasmy.

In addition to a robust laboratory protocol and, preferably, automated rather than manual sample processing, a well-considered data analysis workflow that includes proper procedures for data interpretation and handling is essential to the generation of high quality, error-free mtDNA data for forensic genetic or other purposes. For the development of complete mtGenome haplotypes we recommend adoption of the best practice alignment, nomenclature and reporting guidelines outlined for the production of mtDNA CR data for forensics [27-29]. We also recommend review of the raw electropherogram data by *at least* two scientists and fully electronic data transfer, as described in [13,30]. Further, with the use of a multi-amplicon protocol such as the one presented here, and especially if any manual processing must be performed, we suggest additional post-data production checks to confirm that each complete mtGenome haplotype represents data from a single sample.

## Conclusion

We have developed a high-throughput amplification and sequencing strategy that regularly produces redundant sequence coverage across the entire mtGenome in the first pass of automated data generation. The described workflow, especially when implemented on robotic instrumentation, reduces both the cost of mtGenome sequencing and the opportunities for human error by decreasing the extent of manual sample processing/reprocessing required. As the amplification and sequencing primers were carefully selected based on highly conserved regions of the mtGenome, the protocol works equally well on samples originating from diverse mtDNA haplogroups, yet minimizes the opportunity for non-specific binding that could result in NUMT amplification. DNA input quantities between 50 pg and 1 ng are recommended to maximize first-pass data production success, however high-quality data and complete mtGenome haplotypes can be generated from substantially lower DNA quantities.

This strategy should facilitate more rapid production of the complete mtGenome population reference data needed for future forensic applications, and, when combined with the adoption of best-practice data review and interpretation strategies, ensure that the data sets are of the highest quality possible. In addition, high-quality data developed using this protocol can be utilized comparatively to evaluate mtDNA data produced using various next-generation sequencing chemistries and platforms, an essential first step on the path to eventual implementation of these new technologies in forensics. Finally, the amplification portion of the assay also has clear application as a straight-forward method to enrich samples for mtDNA for next-generation sequencing studies in any discipline.

## Methods

### PCR-amplification

PCR (using the primers listed in Table 1) is performed in a 50 μL total reaction volume using 5 μL GeneAmp 10X PCR Buffer I (Life Technologies, Applied Biosystems), 4 μL GeneAmp® dNTP blend 10 mM (Life Technologies, Applied Biosystems), 2 μL of each 10 μM amplification primer, 3 μL DNA extract, 0.5 μL (2.5 units) AmpliTaq Gold® DNA Polymerase (Life Technologies, Applied Biosystems), and 33.5 μL deionized water. Thermal cycling conditions are: 96°C hold for 10 minutes; 40 cycles of 94°C for 15 seconds, 56°C for 30 seconds, 72°C for 2.5 minutes; and a 72°C hold for 7 minutes. A 96-well plate layout for the simultaneous amplification of eleven samples (plus one negative control per amplicon) is given in Figure 1.

High-throughput amplification of the mtGenome in our laboratory is performed on a liquid-handling instrument (MICROLAB® STARlet, Hamilton Robotics, Reno, NV), utilizing single-use, pre-made tubes of amplification master mix (containing all amplification reagents except enzyme) for each amplicon. The use of pre-made master mixes streamlines the process of amplification set-up for a full 96-well plate, reduces the number of re-amplifications required due to pipetting errors, limits the number of potential causes when an amplification failure occurs, and minimizes the number of freeze-thaw cycles for reagents and primers. For our applications, an amplification master mix is prepared in a 15 mL conical tube using 850 μL GeneAmp® 10X PCR Buffer I (Life Technologies, Applied Biosystems), 680 μL GeneAmp® dNTP blend 10 mM (Life Technologies, Applied Biosystems), 340 μL of each 10 μM amplification primer, and 5695 μL deionized water. Then, 744 μL is aliquoted to each of ten labeled 1.7 mL tubes and stored at -20°C. Just prior to PCR reaction set-up, 8 μL (40 units) AmpliTaq Gold® DNA Polymerase (Life Technologies, Applied Biosystems) is added to the defrosted tube of master mix.

With our semi-automated process, amplification success is assessed by capillary electrophoresis. PCR products are injected directly from the 96-well amplification plate on a QIAxcel Advanced instrument (QIAGEN Inc.), and sizing of the products is performed using the QX alignment marker 50 bp/5 kb and the QX DNA size marker 250 bp-4 kb (QIAGEN Inc.). Alternatively, confirmation that the correct size PCR products were generated could be obtained by gel electrophoresis or another method.

### PCR product purification

Purification of amplification products prior to sequencing is performed enzymatically, using 10 μL Exonuclease I and 5 μL recombinant Shrimp Alkaline Phosphotase (Affymetrix, USB, Cleveland, OH) per 50 μL PCR product. For purification of a full 96-well plate of samples, a master mix of 1100 μL Exonuclease I and 550 μL recombinant Shrimp Alkaline Phosphotase is prepared, and 15 μL of the master mix is manually pipetted to each sample PCR product. Negative controls in column 12 of the 96-well plate are not purified. Thermal cycling conditions are 37°C for 20 minutes followed by 90°C for 20 minutes.

### Sanger sequencing

Each mtGenome is sequenced in a total of 135 reactions using 127 unique primers. The sequencing primers used for each of the eight mtGenome amplicons are listed in Table 2. Sequencing reactions include 8 μL deionized water; 6 μL dilution buffer (400 mmol/l TRIS, 10 mmol/l MgCl2, pH 9.0); either 2 μL BigDye® v1.1 (Life Technologies, Applied Biosystems) for forward sequencing reactions, or 1.5 μL BigDye® v1.1 and 0.5 μL dGTP BigDye® v1.1 (Life Technologies, Applied Biosystems) for reverse sequencing reactions; 2 μL sequencing primer at 10 μM; and 2 μL PCR product for a total reaction volume of 20 μL. Thermal cycling conditions are as follows: 96°C hold for 1 minute, followed by 25 cycles of 96°C for 15 seconds, 50°C for 5 seconds, and 60°C for 2 minutes.

**Figure 9:**
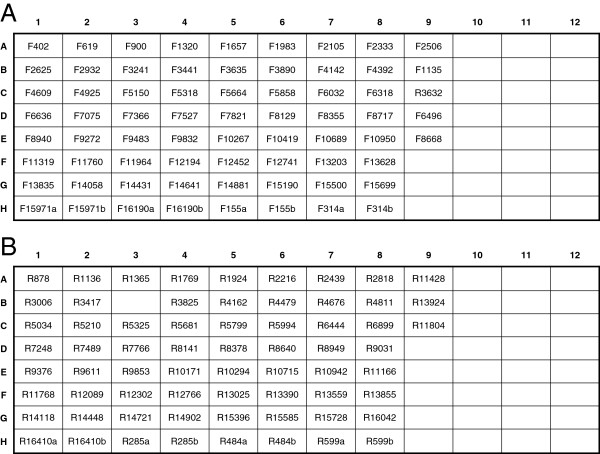
**Forward and reverse primer plate maps for high-throughput processing.** 96-well plate layouts for single-use forward **(A)** and reverse **(B)** sequencing primer plates. Primer plates are prepared robotically to contain 50 μL of each 10 μM primer.

**Figure 10:**
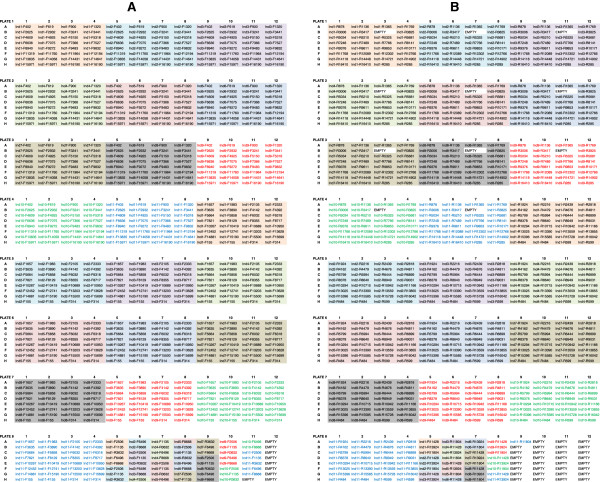
**Sequencing plate maps for high-throughput processing.** 96-well plate layouts for sequencing eleven complete mtGenomes in two sets (forward, **A**; and reverse, **B**), for a total of sixteen sequencing plates per eleven mtGenomes. The eleven different samples are indicated by Ind1, Ind2, etc. and are color-coded, and the primer for each plate well is listed. Empty wells are noted. These plate layouts represent one strategy for high-throughput sequencing, and were specifically designed for efficient pipetting on our laboratory’s liquid handling instruments (MICROLAB® STARlet and STARplus, Hamilton Robotics). A different layout may be more appropriate/more efficient with other instrumentation.

For high-throughput sequencing of eleven amplified samples at a time in our laboratory, all pipetting steps are performed on a liquid-handling instrument (MICROLAB® STARplus, Hamilton Robotics) using a master mix of sequencing reagents and pre-made, single-use primer plates. Sequencing reaction set-up is performed in two sets: one set for the forward sequencing primers, and the second set for the reverse sequences. To ensure sufficient volume for instrument pipetting, sequencing master mixes are prepared in 15 mL conical tubes using 6958 μL deionized water, 5219 μL dilution buffer, and 1740 μL BigDye® v1.1 (for forward sequencing; 1281 μL BigDye® v1.1 plus 427 μL dGTP BigDye® v1.1 is used instead for reverse sequencing). Primer plates (also prepared robotically) include 50 μL of each 10 μM primer according to the plate layouts in Figure 9. Sequencing plate maps (eight forward and eight reverse, for a total of sixteen) for the described high-throughput process are given in Figure 10.

### Sequence product purification

Sequence product purification is performed via gel filtration. For our high-throughput process, Performa DTR V3 96-well short plates (Edge Biosystems, Gaithersburg, MD) are used, and purification steps are performed in two eight-plate batches. Performa plates are first manually centrifuged at 850 g for two minutes to remove some liquid, as per the manufacturer’s recommendation. Subsequently, pipetting from the sequencing product plates to the prepared Performa plates is performed robotically, then filtration into new, barcoded 96-well plates is accomplished by manual centrifugation for 5 minutes at 850 g.

### Sequence detection and analysis

Purified sequence products are evaporated by heated vacuum centrifugation then resuspended in 10 μL Hi-Di™ Formamide (Life Technologies, Applied Biosystems). For our high-throughput process, sequence detection is performed by capillary electrophoresis on a 3730 Genetic Analyzer (Life Technologies, Applied Biosystems) using a 50 cm array, the FastSeq instrument protocol (FastSeq50_POP7 run module) and the SeqAnalysis Fast analysis protocol (Basecaller_3730POP7RR) with the default instrument settings. Post-detection, raw signal data is initially processed on the 3730 Genetic Analyzer computer using Sequencing Analysis v5.3.1 (Life Technologies, Applied Biosystems) with the spacing parameters set to 12.0.

Trimming, assembly and review of the processed electropherograms is performed in Sequencher version 4.8 or 5.0 (Gene Codes Corporation, Ann Arbor, MI). Sequences are aligned to the rCRS [22]. For our purposes, and in accordance with current requirements for publication of mtDNA data sets [31], at least two high-quality, high-resolution sequences covering every mtGenome position are required for development of a complete mtGenome haplotype.

### Notes on instrumentation

While we currently utilize Hamilton Robotics liquid handling instruments (MICROLAB® STARlet and STARplus) for pre and post-PCR pipetting, portions of the assay development and developmental validation were performed on a Tecan Genesis® 2000 workstation (Tecan Group Ltd., San Jose, CA). The described workflow could be implemented on any fit for purpose liquid handling instruments, and the plate layouts (such as those depicted in Figure 10 for sequencing) modified according to instrument set-up and desired throughput.

Thermal cycling steps in this protocol have been performed with equal success on a variety of 96-well machines in our laboratory, including GeneAmp® PCR System 9700 and Veriti® instruments (Life Technologies, Applied Biosystems), TRobot thermal cyclers (Biometra GmbH, Goettingen, Germany), and PTC-0200 DNA Engine instruments (MJ Research, Inc., Waltham, MA). The described cycling parameters should thus be appropriate for implementation on most thermal cyclers with little, if any, optimization needed.

## Competing interests

The authors declare that they have no competing interests.

## Authors’ contributions

EAL designed the primers, performed the developmental work and testing, analyzed the data, helped troubleshoot the protocol and helped prepare the manuscript. MKS helped design the strategy, helped troubleshoot the protocol, helped develop the custom robotic instrument methods, and helped analyze the data. KSA helped design the strategy, designed the robotic instruments, developed the custom robotic instrument methods, and helped analyze the data. JAI helped conceptualize the work, helped design the strategy, and helped prepare the manuscript. RSJ conceptualized the work, designed the strategy, managed protocol troubleshooting, helped design the robotic instrument methods, helped analyze the data, and prepared the manuscript. All authors read and approved the final manuscript.

## Authors’ information

MKS, KSA and RSJ (and, formerly, EAL and JAI) are American Registry of Pathology contractors supporting the Armed Forces DNA Identification Laboratory, Armed Forces Medical Examiner System. RSJ is also a Ph.D. candidate at the University of Maryland, College Park.
